# Enantioselctive Syntheses of Sulfur Analogues of Flavan-3-Ols

**DOI:** 10.3390/molecules15085595

**Published:** 2010-08-13

**Authors:** Pradeep K. Sharma, Min He, Jurjus Jurayj, Da-Ming Gou, Richard Lombardy, Leo J. Romanczyk Jr., Hagen Schroeter

**Affiliations:** 1 Chemical Process Research &amp; Development and Analytical Development, Johnson Matthey Pharmaceutical Materials Inc., 25 Patton Road, Devens, MA 01434, USA; 2 Chemical Process R&amp;D and CMC Regulatory, ARIAD Pharmaceuticals Inc., Cambridge, MA 02139, USA; 3 MARS Incorporated, 6885 Elm Street, McLean, VA 22101, USA

**Keywords:** flavan-3-ols, 5,7-dideoxythiocatechin, 5,7-dideoxythioepicatechin, thiocatechin, thioepicatechin, asymmetric dihydroxylation

## Abstract

The first enantioselective syntheses of sulfur flavan-3-ol analogues **1**–**8 **have been accomplished, whereby the oxygen atom of the pyran ring has been replaced by a sulfur atom. The key steps were: (a) Pd(0) catalyzed introduction of –S *t-*butyl group, (b) Sharpless enantioselective dihydroxylation of the alkene, (c) acid catalyzed ring closure to produce the thiopyran ring, and (d) removal of benzyl groups using *N,N*-dimethylaniline and AlCl_3_. The compounds were isolated in high chemical and optical purity.

## 1. Introduction

**Figure 1:**
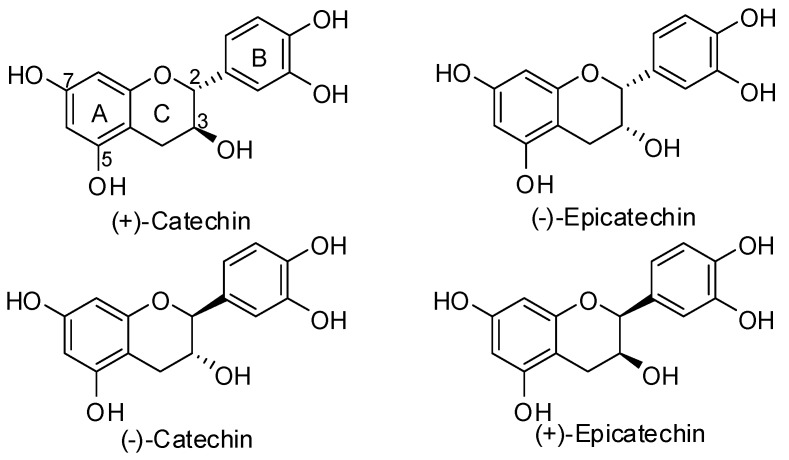
Structures of catechin and epicatechin enantiomers.

Oligomeric and polymeric proanthocyanidins are some of the most ubiquitous groups of all the plant phenolics [1] and their health benefits are well known. A large number of *in vitro* studies have characterized flavanols as powerful antioxidants capable of efficient scavenging of both reactive oxygen and reactive nitrogen species [2,3,4,5,6,7,8]. The mechanism of their action as radical scavengers involves the donation of a hydrogen atom and/or electron to stabilize the radical species [9]. Until recently, the ability of flavanols, and indeed other flavonoids, to act as classical H-donating antioxidants was believed to underlie many of their reported health benefits [10,11,12,13,14,15,16]. However, it is now clear that their ultimate antioxidant potential, and indeed their resulting potential *in vivo* bioactivity is dependent on the absorption, metabolism, distribution, reducing properties of the resulting metabolites, and excretion of these compounds within the body after ingestion. An understanding of the processes involved in the absorption and distribution of flavonoids is essential to determine their potential bioactivities *in vivo* and their overall significance in disease prevention [17]. The building blocks for most of these oligomeric and polymeric proanthocyanidins are the flavan-3-ols (+)-catechin and (-)-epicatechin, whose stereochemistry allows for a large array of structural possibilities [1]. In nature, (+)-catechin is widely distributed, whereas (-)-epicatechin, (-)-catechin, and (+)-epicatechin are much less abundant (Figure 1).

This structural diversity of polyphenols is a subject of interest because of their varied biological activities [18,19,20,21,22,23]. Included are an interesting group of sulfur conjugated flavan-3-ols that are derived from cysteine and cysteamine [24,25,26,27,28,29,30,31]. Replacement of oxygen with sulfur is particularly appealing because of the isovalent and isosteric relationship between sulfur and oxygen with minimal structural disruption and possibly impacting the activity and the metabolism of the molecule. Sulfur exists in a variety of oxidation states including -2, +4, and +6. The sulfur can undergo single oxidation to the sulfoxide or it may be further oxidized to sulfone. The replacement of oxygen in the pyran ring with sulfur might offer profound changes in the pharmacological action of the molecules. We are unaware of any reports which describe the occurrence of thiocatechins or its analogues, whereby the oxygen in the pyran ring is replaced with sulfur, although the synthesis of thioflavones and their methoxy derivatives have been described [31,32]. Wang *et al*. [33] also reported two novel sulfur containing flavanols isolated from the leaves of *Glycosmis montana*. Additionally, Lonano *et al*. [34] has reported that 4β-(*S*-cysteinyl)epicatechin-3-*O*-gallate has a free radical scavenging capacity as strong as that of (-)-epigallocatechin gallate and causes significant S-phase cell cycle arrest in certain cell lines at doses higher than 100 μM. The other cysteinyl compounds do not affect normal cell cycle distribution. The gallate derivatives also induce apoptosis in melanoma cells more strongly than the other derivatives and (-)-epicatechin. The gallate compound seems to trigger nuclear condensation and fragmentation, which was confirmed by DNA laddering. However, they do not induce apoptosis in keratinocytes (HaCaT). 

**Figure 2:**
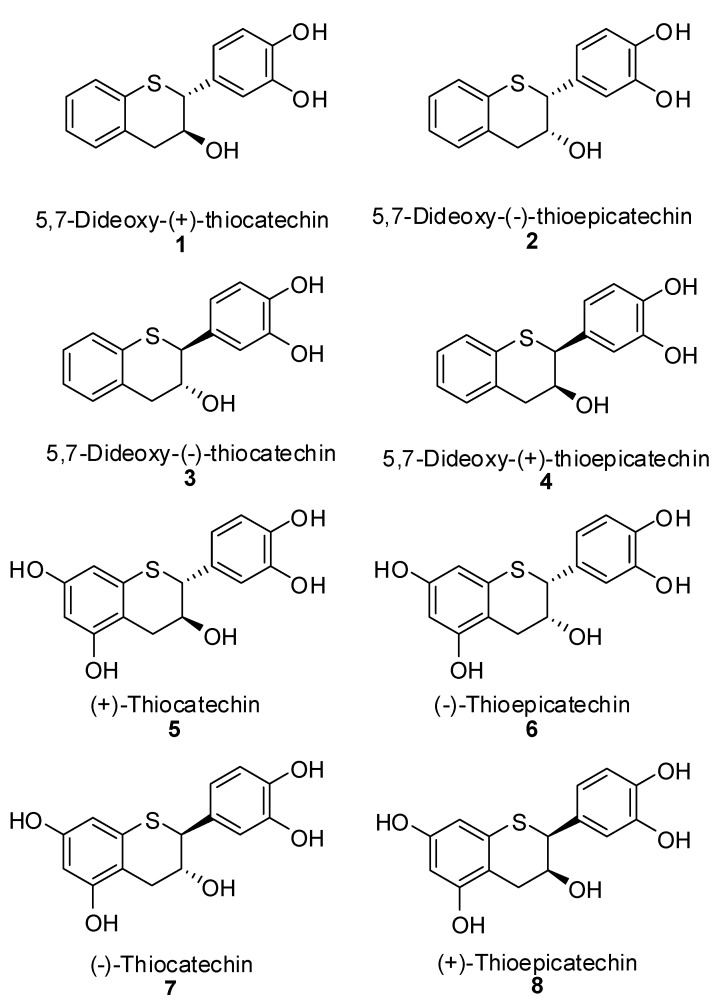
Structures of analogues of catechin and epicatechin enantiomers.

In this report, we wish to describe the first syntheses of sulfur analogues for all four isomers and A-ring dideoxy analogues of naturally occurring catechin. These are 5,7-dideoxy-(+)-thiocatechin (**1**), 5,7-dideoxy-(-)-thioepicatechin (**2**), 5,7-dideoxy-(-)-thiocatechin (**3**), 5,7-dideoxy-(+)-thioepicatechin (**4**) and fully substituted analogues namely (+)-thiocatechin (**5**), (-)-thioepicatechin (**6**), (-)-thiocatechin (**7**), and (+)-thioepicatechin (**8**), as depicted in Figure 2.

## 2. Results and Discussion

**Figure 3:**
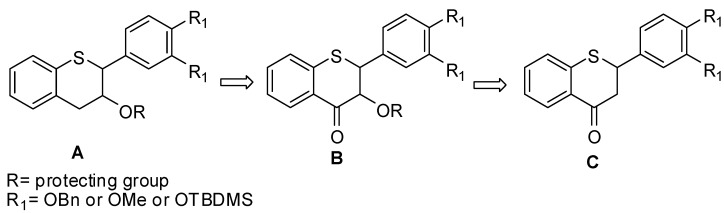
Initial retrosynthetic approach.

Our aim was to initially develop a methodology for the synthesis of **1** to **4** and then apply the same methodology for the analogues **5** to **8**. The retro-synthetic approach for the construction of **1** is depicted in Figure 3, which we envisioned as a direct approach. We contemplated the use of **C** as a starting material where enantio-selective α-hydroxylation of the 4-position ketone could be achieved via Davis methodology [35,36] or by converting the ketone *in situ* to a silyl enol ether followed by Sharpless asymmetric dihydroxylation [37]. Initially, we explored the methodology on a commercially available model compound, namely phenylthiochroman-3-one (R_1_=H). Thus, attempts to introduce the 3-position hydroxyl group to phenylthiochroman-3-one using Mn(OAc)_3_•2H_2_O either in benzene or toluene at reflux or in a mixture of benzene/glacial AcOH or CH_2_Cl_2_ with or without glacial AcOH resulted in a complex and inseparable mixture [38]. 

**Figure 4:**
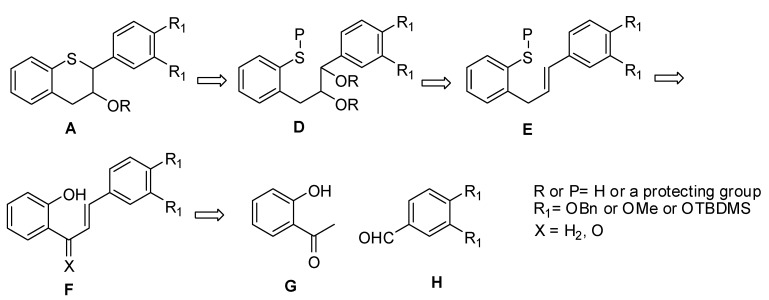
Modified retrosynthetic approach.

Alternatively, attempts to react phenylthiochroman-3-one with TMSCl or TBDMSCl in the presence of Et_3_N/DMAP in MTBE or Et_3_N/DMAP in CH_2_Cl_2_ at ambient temperature *in situ* followed by reaction under Sharpless asymmetric dihdyroxylation did not produce the desired compound. In all the attempts, the starting material was recovered. Having been unsuccessful in the direct approach for the construction of the desired compounds via the introduction of hydroxyl group α to the ketone, we modified our approach as depicted in Figure 4.

In Figure 4, the challenge resides in converting the phenolic group to a suitably protected thiol group, which could then be deprotected to form the desired intermediate **A**. An additional challenge would be to retain the stereochemistry at the benzylic 2-position under the deprotection conditions. The use of the benzyl group was chosen to protect the phenolic groups because of the ease of removal under mild conditions.

### 2.1. Synthesis of *16* and *17*

**Scheme 1:**
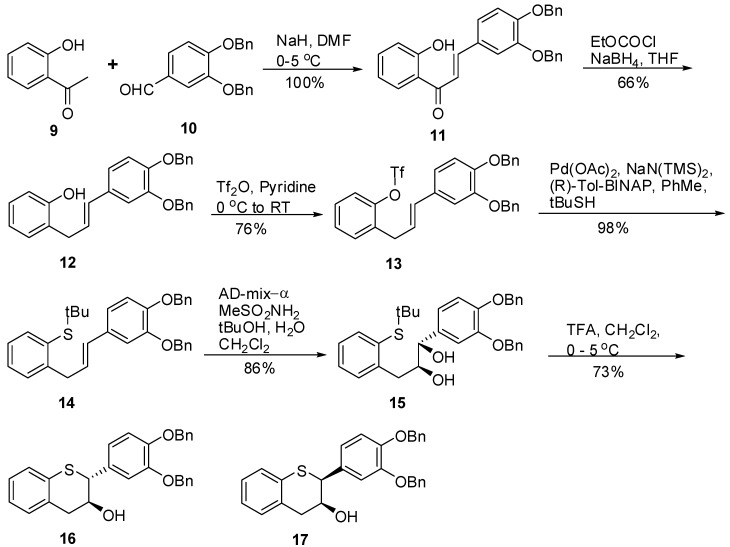
Synthesis of **16** and **17**.

In Scheme 1, the base catalyzed condensation between 2-hydroxyacetophenone **9** and 3,4-bis-(benzyloxy)benzaldehyde **10** furnished the chalcone **11** in quantitative yield via a Claisen-Schmidt reaction. Selective reduction of the keto group of **11** with NaBH_4_ and CeCl_3_•7H_2_O in a mixture of THF and water resulted in the desired compound **12** in low yield after chromatography [39]. Alternatively, when **11** was subjected to treatment with ethyl chloroformate in the presence of Et_3_N in THF at 0 °C, the corresponding ethyl carbamate intermediate was formed and reacted with NaBH_4_ in aqueous media after removal of the salts. After workup and purification, **12** was furnished in 66% yield [40].

The reaction of **12** with Tf_2_O in pyridine at ambient temperature yielded **13** in 76% yield after chromatography. The use of other bases such as Et_3_N, DMAP or DIPEA in THF or CH_2_Cl_2_ did not result in complete reaction. We envisioned the introduction of an –STIPS group [41] where the TIPS could be removed under mild acidic conditions or by nBu_4_NF to generate the thiol. Our attempts to effect Pd catalyzed coupling of TIPSSH with triflate **13** using Pd(PPh_3_)_ 4_, Pd(OAc)_ 2_, (*R*)-Tol-BINAP and TIPSH in the presence of a base (NaH or NaN(TMS)_2_) in toluene did not produce the desired compound and the starting material was recovered. Alternatively, the introduction of an –S *t-*butyl group was accomplished using the methodology reported by McWilliams *et al.* [42]. Under the conditions, triflate **13** was treated with NaS*t*-Bu or KS*t*-Bu (generated *in situ* by reacting HS*t*-Bu with NaHDMS or KHDMS at ambient temperature in the presence of Pd(OAc)_ 2_, (*R*)-Tol-BINAP in toluene at 100 °C for 18h to produce **14** in 98% yield after chromatography. Attempts to replace Pd(OAc)_2_, (*R*)-Tol-BINAP with Pd(PPh_3_)_4_ under similar conditions did not produce the desired compound. Sharpless asymmetric dihydroxylation of **14** with AD-mix-α and CH_3_SO_2_NH_2_ in the presence of *^t^*BuOH, H_2_O and CH_2_Cl_2_ at 0 °C resulted in **15** in 86% yield [43]. Attempts to replace CH_2_Cl_2_ with acetone or toluene did produce **15** but in low yield and *ee*. Literature precedence and conversion of **15** to **16** established the stereochemical assignment of **15**. The *ee* of **15** was 100% as determined by chiral HPLC. The dihydroxylation of **14** also furnished ~5% of a sulfoxide by product, which was easily removed by chromatography. Having the key intermediate **15**, we focused our attention at the removal of the *tert*-butyl group followed by cyclization under acidic conditions. The treatment of **15** with excess TFA in CH_2_Cl_2_ at ambient temperature for 24 h resulted in a complex mixture. However, LCMS indicated the presence of only a trace of the desired compound. These results revealed product formation, but additional by products were also formed. LCMS showed that *t*-butylation of the oxygenated phenyl group was a side reaction which prompted the examination of cationic scavengers. Reacting **15** with TFA in CH_2_Cl_2_ in the presence of *i*Pr_3_SiH or anisole as scavengers at 0 °C resulted in the formation of two non-polar major products as determined by HPLC. However, when the workup was performed at ambient temperature, multiple products were obtained probably the result of product decomposition. A modification was made in the reaction work up conditions where **15** was treated with TFA in CH_2_Cl_2_ at 0 °C for 24h. Under these modified conditions, the reaction was quenched with an equimolar amount of Et_3_N at 0 °C followed by the addition of cold water (<5 °C) before bringing the reaction mixture to ambient temperature for thin layer separation. The crude product was purified by chromatography producing **16** and **17** in 93:7 ratio, respectively. The enantiomeric excess of **16** and **17** were determined to be 96% and 100% respectively by chiral HPLC analysis. Compound **16** having *trans*-2,3-orientation was isolated as the major component along with the *cis*-isomer **17** as a minor product. The formation of **17** under these conditions suggested that the activated benzylic 2-position in the sequence could not maintain its stereochemical integrity, thus producing **17** as a minor diastereomer. 

### 2.2. Proposed mechanism for the formation of *16 *and *17*

**Scheme 2:**
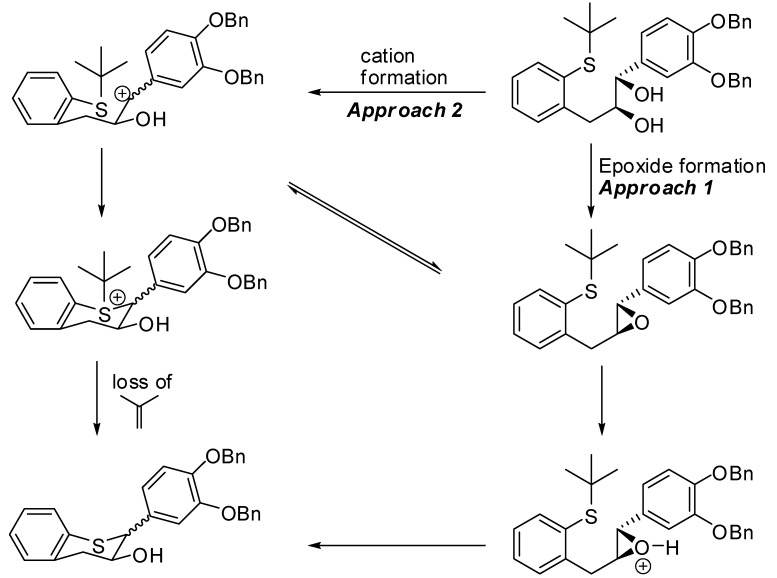
Proposed mechanism of the cyclization of **15**.

The proposed mechanism for the formation of **16** and **17** is outlined in Scheme 2. The benzylic carbocations generated *in situ* might form the epoxide which is followed by ring closure (Approach 1) in the presence of an acid. Alternatively, the attack of the sulfur atom to close the ring (Approach 2) followed by loss of the *tert*-butyl group as isobutene would lead to product formation. Thus, the carbocation generated at benzylic 2-position would lose its stereochemical integrity and thus form the minor isomer **17**. Formation of both catechin and epicatehin derivatives is explicable in terms of the generation of an incipient benzylic 2-position carbocation *via* protonation of the benzylic alcohol functionality, and subsequent S_N_1 cyclization that leads to the predominant formation of the thermodynamically more stable *trans*-isomer along with the *cis* isomer. If the formation of the diastereomer occurs via carbocation scrambling, then the diastereomeric ratio would be 1:1. Note that other mechanisms are possible for the formation of the diastereomers. No kinetic studies were performed at this time to determine the stability of the benzylic 2-position carbocation. However, from the ratio of the major and minor isomer it was concluded that temperature might play an important role [44,45,46].

The use of other acids such as 6N HCl or CH_3_SO_3_H in either CH_2_Cl_2_ or THF in place of TFA at 0 °C were investigated and resulted in only < 50% conversion after 48 h. 

### 2.3. Synthesis of *19* and *20*

**Scheme 3:**
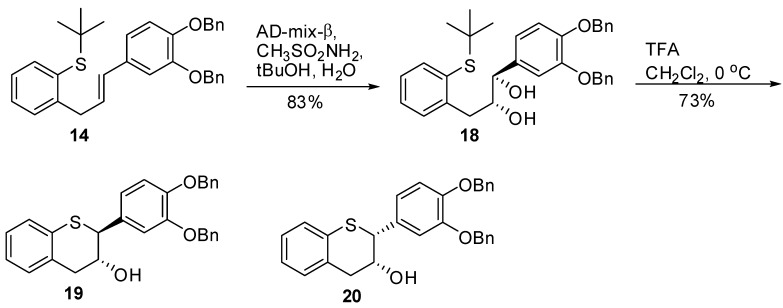
Synthesis of **19** and **20**.

For the synthesis of **19** and **20** (*Scheme 3*), alkene **14** was reacted with AD-mix-b under Sharpless asymmetric dihydroxylation conditions giving **18** in 83% yield, and 100% *ee*.

Compound **18** has a similar NMR spectrum to **15** but opposite optical rotation. The presence of ~5% sulfoxide, which was efficiently removed by chromatography, was observed during the formation of **18**. The treatment of **18** with TFA in CH_2_Cl_2_ at 0 °C resulted in the major **19** and a minor diasteromer **20** after chromatographic purification. The enantiomeric excess of **19** and **20** were found to be 99% and 99.5%, respectively.

### 2.4. Synthesis of *1,* *2,* *3,* and *4*

**Scheme 4:**
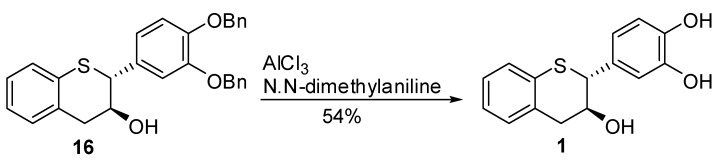
Debenzylation of **16**.

Once the synthesis of the penultimate intermediates **16**, **17**, **19**, and **20** were accomplished, our attention was turned to optimizing the debenzylation conditions. The catalytic hydrogenation of **16** with 20% Pd/C or 10% Pd(OH)_2_/C in CH_3_OH, EtOAc or CH_3_OH/EtOAc (1/4, v/v) resulted in an incomplete reaction. Increasing catalyst loading, extending the reaction time (up to 72 h) or increasing the hydrogen pressure (up to 50 psi) did not change the course of the reaction. Use of Pd black along with 1,4-cyclohexadiene with or without the presence of a scavenger (*N,N*-dimethylaniline) or 5%Pd/Al_2_O_3_ at 15 psi H_2_ pressure in EtOAc/CH_3_OH (3/2, v/v) did result in an incomplete reaction. The reaction mixture consisted of debenzylated product, partially benzylated intermediates, and the starting material. The incomplete reaction was thought to be due to the poisoning of the catalyst in the presence of the sulfur atom in the molecule. A non-catalytic deprotection method using BCl_3_ with nBu_4_NI was not successful. Finally, compound **16** was treated with AlCl_3_ at 0 °C in the presence of *N,N-*dimethylaniline in CH_2_Cl_2_ to produce **1** in 54% yield after purification by preparative HPLC [47] (Scheme 4).

Under similar conditions, **17**, **19**, and **20** produced **2**, **3**, and **4** respectively in 50 to 64% yield. The *ee* of **1** to **4** were further determined by preparing their peracetylated derivatives. It was found that no loss in optical purity could be observed (≥98% *ee*) for these compounds during the deprotection and isolation stages of preparation. These isomers were found to have similar optical rotation when compared to natural catechin and epicatechin enantiomers.

### 2.5. Synthesis of *27* and *28*

**Scheme 5:**
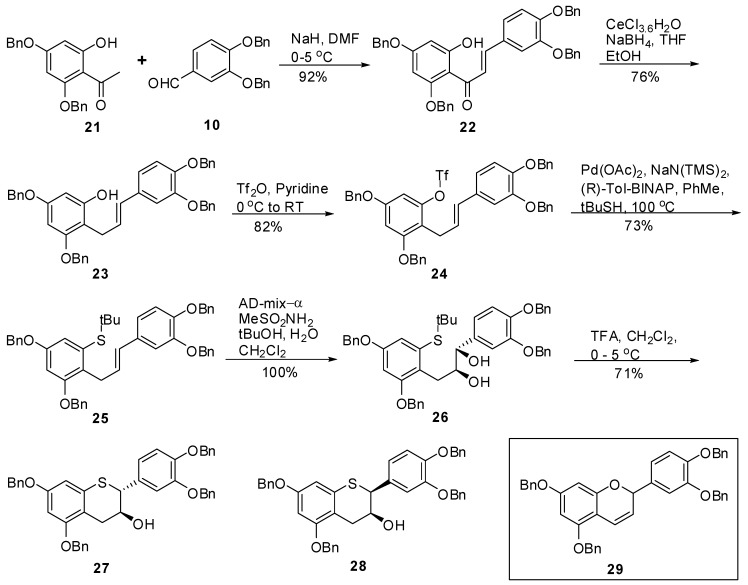
Synthesis of **27** and **28** intermediates.

Encouraged by the successful synthesis of **1** to **4**, we then pursued the synthesis of the sulfur catechin analogues and their epimers, **5** to **8**. Our synthetic approach for these compounds is depicted in Scheme 5.

The base catalyzed condensation between **21** and **10** produced chalcone **22 **via Claisen-Schmidt reaction in 92% yield, and >98% AUC purity. Attempts to reduce the conjugated ketone of **22** by ethyl chloroformate and NaBH_4_ either at 0 °C or at ambient temperature did not produce **23** and the starting material was recovered. Luche reduction of **22** at 0 to 5 °C resulted in **23** in 83% yield after chromatography. It was essential to perform the reaction at low temperature [39] to avoid the formation of the cyclic byproduct **29**. Reaction of **23** with Tf_2_O in pyridine at ambient temperature produced triflate **24** in good yield after chromatography. The triflate **24** was subjected to Pd(OAc)_2_ and (*R*)-Tol-BINAP in the presence of *t*-BuSH and NaN(TMS)_2_ in toluene at 100 °C to give **25**. The asymmetric dihydroxylation of **25** under Sharpless conditions using AD-mix-α along with methane sulfonamide in a mixture of *tert*-butanol, water and CH_2_Cl_2_ produced **26** in 86% yield. It was again observed that ~5% of corresponding sulfoxide was obtained, which was removed during purification by chromatography. When **26** was subjected to TFA in CH_2_Cl_2_ at 0–5 °C for 24 h, **27** and **28** were obtained as the major and minor diastereomers, respectively after chromatography in 96% *ee*.

### 2.6. Synthesis of *31* and *32*

**Scheme 6:**
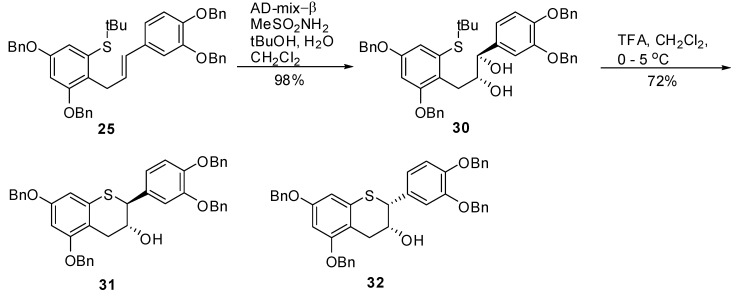
Synthesis of intermediates **31** and **32**.

Having established the syntheses of **27** and **28** for **5** and **8,** respectively alkene **25** was subjected to AD-mix-β along with methane sulfonamide in a mixture of *tert*-butanol, water and CH_2_Cl_2_ producing **30** in 83% yield (Scheme 6). The diol **30** has the same spectroscopic properties as **26** but opposite optical rotation. 

The reaction of **30** with TFA in CH_2_Cl_2 _at 0–5 °C for 24 h resulted in the major and minor diastereomer **31** and **32** respectively after chromatography, and in >96% *ee*.

### 2.7. Synthesis of *5, 6, 7,* and *8*

The treatment of a cold solution of **27**, **32**, **31** and **28** in CH_2_Cl_2_ with an excess of AlCl_3_ in the presence of *N,N-*dimethylaniline as a scavenger resulted in **5**, **6**, **7**, and **8,** respectively after HPLC purification in 50 to 65% yield. A comparison of optical rotations of newly synthesized thiocatechin and thioepicatechin enantiomers (**1** to **8**) against naturally occurring catechin and epicatechin enantiomers are summarized in Table 1.

**Table 1:** Optical rotations of the sulfur analogues of flavan-3-ols.

Compounds	Optical rotations*
(+)-Catechin	+ 56.6
5,7-Dideoxy-(+)-thiocatechin (**1**)	+ 22.2
(+)-Thiocatechin (**5**)	+ 32.2
(-)-Epicatechin	- 33.9
5,7-Dideoxy-(-)-thioepicatechin (**2**)	- 28.6
(-)-Thioepicatechin (**6**)	- 28.9
(-)-Catechin	- 34.8
5,7-Dideoxy-(-)-thiocatechin (**3**)	- 21.3
(-)-Thiocatechin (**7**)	- 28.6
(+)-Epicatechin	+ 37.7
5,7-Dideoxy-(+)-thioepicatechin (**4**)	+ 28.4
(+)-Thioepicatechin (**8**)	+ 29.7

* (*c 1*, acetone).

## 3. Experimental

### 3.1. General

All the solvents were purchased from Aldrich Chemical Company in Sure/Seal™ bottles and were used as received. ^1^H-NMR spectra were recorded on a 300 MHz Bruker whereas ^13^C-NMR spectra were recorded on a 75 MHz Bruker NMR and TMS was used as an internal standard. Specific rotations were determined for solutions by irradiating with the sodium D line (λ= 589 nm) using a Perkin Elmer 341 polarimeter: specific rotation, [α]_D_ values are given in units 10^-1^deg•cm^2^g^-1^ where the concentration c is given in g/100 mL. The chemical purity (Method A) was determined by standard HPLC (containing a PDA detector) using a Phenomenex Synergi 4 μ Fusion-RP 80 Å (150 mm × 4.6 mm) column at wavelength of 280 nm, using a gradient of 5 to 90% of acetonitrile (containing 0.01% TFA) with water (containing 0.01% TFA) up to 20 min, the column temperature was 25 °C, and the flow rate was 1 mL/min. The enantiomeric purity (Method B) was determined by chiral HPLC equipped with a PDA detector using a Chiralpak AD-RH 5μ (150 mm × 4.6 mm) column. The solvent for isocratic programs was acetonitrile/water (65/35, v/v) containing 0.01% TFA, run time 40 min, detection wavelength was 210 nm, column temperature was 60 °C, and the flow rate was 1 mL/min. 

*(E)-3-(3’,4’-Bis(benzyloxy)phenyl)-1-(2-hydroxyphenyl)prop-2-en-1-one* (**11**). To a suspension of NaH (60% dispersion in oil, 3.82 g, 95.48 mmol, 1.3 eq.) in dry DMF (100 mL) was slowly added **9** (10 g, 73.45 mmol, 1 eq.) keeping the internal temperature ≤ 2 °C throughout the addition. The resulting mixture was stirred at this temperature for 10 minutes. A solution of **10** (23.38 g, 73.45 mmol, 1 eq.) in DMF (100 mL) was added over a period of 20 minutes to the reaction mixture *via* an addition funnel keeping the internal temperature ≤ 2 °C. The resulting red brown solution was stirred at this temperature for an additional 30 minutes before stirring at RT for 4 hours. The reaction mixture was quenched with H_2_O (50 mL) and diluted with EtOAc (500 mL). The organic layer was separated, washed with H_2_O (50 mL), sat. NaHCO_3_ (2 × 50 mL), brine (100 mL), dried (Na_2_SO_4_), filtered and the solvent removed *in vacuo* to afford a yellow solid. The solid was triturated with heptane (100 mL) at RT for 1h and filtered. The solids were dried under high vacuum at RT for 18 h to produce **11** (33.3 g, 100%) as a yellow solid with 100% AUC purity. ^1^H-NMR (CDCl_3_) δ = 5.32 (s, 4H), 6.2 (dd, 2H, *J* = 2.2 and 19Hz), 6.9–7.1 (m, 4H, Ar), 7.2–7.6 (m, 9H, Ar), 7.8–8.0 (m, 4H, Ar); ^13^C-NMR (CDCl_3_) δ = 31.1, 37.2, 47.3, 51.5, 117.4, 118.2, 118.4, 126.3, 127.3, 127.4, 127.5, 127.8, 128.4, 128.7, 129.1, 129.3, 130.2, 131.7, 132.1, 137.4, 138.9, 139.9, 148.6, 149.9, 150.3, 181.6; MS (*m/z*): 437 (M^+^+1); HRMS calcd for C_29_H_24_O_4_ [M+H] 437.1753, Found: 437.1748.

*(E)-2-(3-(3’,4’-Bis(benzyloxy)phenyl)allylphenol* (**12**). To a solution of **11** (12 g, 27.6 mmol, 1 eq.) in THF (100 mL) was added Et_3_N (5 mL, 35.8 mmol, 1.3 eq.) and the mixture cooled to 0 °C. Ethyl chloroformate (3.2 mL, 33 mmol, 1.2 eq.) was slowly added keeping the internal temperature at 0 °C. The resulting mixture was allowed to stir at 0 °C for 1.5 h and the progress of the reaction was monitored by TLC. The reaction mixture was suction filtered to remove the salts and the salts washed with THF (2 × 50 mL). The combined filtrate was added slowly to a cold solution of NaBH_4_ in water at 0 °C over 45 minutes. The resulting mixture was slowly warmed to room temperature and allowed to stir for 18h. The reaction mixture was acidified with 1N HCl (pH ~2 with a pH paper) and extracted with EtOAc (2 × 300 mL). The combined organic layers were dried (MgSO_4_), filtered and the solvent was removed *in vacuo* to afford an oil. The crude product was purified by silica gel chromatography (5–10% ethyl acetate in heptane) to afford **12** (7.7 g, 66%) as a colorless oil with 100% AUC purity. ^1^H-NMR (CDCl_3_) δ = 3.58 (d, 2H, *J* = 6.0 Hz), 5.2 (s, 4H), 6.1–6.25 (m, 1H), 6.33 (d, 1H, *J* = 8.4 Hz), 6.8–7.0 (m, 4H), 7.2–7.6 (m, 13H); ^13^C-NMR (CDCl_3_) δ = 34.0, 71.5, 71.9, 71.7, 77.5, 112.9, 115.3, 115.8, 115.9, 119.9, 120.8, 120.9, 125.8, 126.3, 127.3, 127.4, 127.8, 127.8, 127.8, 128.2, 128.5, 130.4, 131.4, 131.1, 131.4, 137.2, 148.6, 148.2, 154.1; MS (*m/z*) = 423.1 (M^+^+1); HRMS calcd for C_29_H_26_O_3_ [M+H] 423.1962, Found: 423.1959

*(E)-2-(3-(3’,4’-Bis(benzyloxy)phenyl)allyl)phenyl trifluoromethane sulfonate* (**13**). To an ice cold solution (<5 °C) of **12** (4 g, 9.46 mmol, 1 eq.) in dry pyridine (40 mL) was slowly added Tf_2_O (3.22 g, 11.4 mmol, 1.2 eq.). The resulting mixture was allowed to warm to RT and allowed to stir for 18h. The reaction mixture was diluted with EtOAc (200 mL), washed with 1N HCl (4 × 100 mL), brine (100 mL), dried (Na_2_SO_4_), filtered and the solvent was removed *in vacuo* to give an oil. The oil was purified by silica gel chromatography (5-10% ethyl acetate in heptane) to give **13** (4 g, 76%) as a colorless oil with 100% AUC purity. ^1^H-NMR (CDCl_3_) δ = 3.6 (d, 2H, *J *= 6.3 Hz), 5.2 (s, 4H), 6.0–6.15 (m, 1H), 6.33 (d, 1H, *J* = 8.4 Hz), 6.8 (s, 2H), 7.0 (s, 1H), 7.2–7.6 (m, 14H); HRMS calcd for C_30_H_25_F_3_O_5_S [M+H] 555.1455, Found: 555.1451

*(E)-2-(3-(3’,4’-Bis(benzyloxy)phenyl)allyl)phenyl-(tert-butyl)sulfane* (**14**). To a degassed solution of **13** (8 g, 14.43 mmol, 1 eq.) in dry toluene (80 mL) was added Pd(OAc)_2_ (0.194 g, 0.866 mmol, 0.06 eq.) and (*R*)-Tol-BINAP (0.685 g, 1.01 mmol, 0.07 eq.) at RT. The resulting reaction mixture was degassed again for an additional 15 minutes at RT. In a separate flask, a solution of NaN(TMS)_2_ (0.6M solution in toluene, 33.7 mL, 202.2 mmol, 1.4 eq.) was added slowly to *t*-BuSH (2.3 mL, 2.2 mmol, 1.4 eq.) in dry toluene (10 mL) at RT and the mixture stirred at RT under N_2_ for 15 minutes. This solution was transferred under N_2_ to the above solution. The red colored solution was heated at 100 °C under N_2_ for 18h. The reaction mixture was diluted with H_2_O (100 mL) and EtOAc (100 mL). The organic layer was separated, washed with brine (50 mL), dried (Na_2_SO_4_), filtered and the solvent was removed *in vacuo* to give a red viscous oil. The oil was purified by silica gel chromatography (5% EtOAc in heptane) to afford **14** (6.98g, 98%) as a yellow viscous oil with 99.8% AUC purity. ^1^H-NMR (CDCl_3_) δ = 1.22 (s, 9H), 3.88 (d, 2H, *J* = 6.7 Hz), 5.12 (s, 4H), 6–6.2 (m, 1H), 6.3 (d, 1H, *J* = 6.7 Hz), 6.77 (s, 2H), 7.0 (s, 1H), 7.1–7.6 (m, 14H); ^13^C-NMR (CDCl_3_) δ = 31.2, 37.9, 47.2, 71.5, 112.1, 115.4, 119.9, 126.2, 127.3, 127.4, 127.7, 127.8, 127.9, 128.5, 128.6, 129.2, 129.9, 130.6, 131.7, 132.1, 137.4, 138.9, 143.8, 145.1, 146.4; MS (*m/z*) = 495 (M^+^+1), 439.3 (M^+^-*t*-Bu); HRMS calcd for C_33_H_34_O_2_S [M+H] 495.2358, Found: 495.2353

*(1S,2S)-1-(3’,4’-Bis(benzyloxy)phenyl)-3-(2-(tert-butylthio)phenyl)propane-1,2-diol* (**15**). A suspension of AD-mix-α (18g) in *^t^*BuOH/H_2_O (60 mL, 1/1, v/v) was stirred at RT for 15 minutes until a clear yellow solution was obtained followed by the addition of a solution of **14** (3.6 g, 7.29 mol, 1 eq.) in CH_2_Cl_2_ (15 mL). The resulting reaction mixture was cooled to 0 ºC with stirring. Then, MeSO_2_NH_2_ (0.833 g, 8.76 mmol, 1.2 eq.) was added and the mixture stirred at this temperature for 24h. The reaction was quenched by the addition of 10% Na_2_S_2_O_3_ (50 mL) and then extracted with CH_2_Cl_2_ (2 × 100 mL). The combined organic layers were washed with H_2_O (2 × 50 mL), brine (1 × 75 mL), dried (Na_2_SO_4_), filtered, and the solvent removed *in vacuo* to afford the crude product. The crude product was purified by silica gel chromatography using 20% to 30% EtOAc/heptane to produce **15** (3.3g, 86%) as an oil with 100% AUC purity. [α]^20^_D _= +34. 9 (*c 1*, acetone); ^1^H-NMR (CDCl_3_) δ = 1.15 (s, 9H), 2.21 (d, 1H, *J = *4.45 Hz), 2.82 (dd, 1H, *J* = 9, 9.5 Hz), 2.88 (d, 1H, *J* = 4.5 Hz), 3.07 (dd, 1H, *J* = 4 Hz), 3.81-3.9 (m, 1H), 4.4 (dd, 1H, *J* = 3.4 Hz), 5.13 (s, 2H), 5.18 (s, 2H), 6.8 (s, 2H), 7.03 (s, 1H), 7.1–7.52 (m, 14H); ^13^C-NMR (CDCl_3_) δ = 30.9, 38.5, 47.6, 71.3, 71.5, 113.9, 115.1, 120.4, 126.6, 127.3, 127.5, 127.7, 127.9, 128.5, 129.1, 130.9, 132.4, 134.8, 137.3, 137.4, 139.1, 143.8, 148.8, 149.2; Optical purity = 100% *ee. *HRMS calcd for C_33_H_36_O_4_S [M+H] 529.2412, Found: 529.2338.

*3’,4’-Bis(benzyloxy)-5,7-dideoxy-(+)-thiocatechin* (**16**) *and 3’,4’-Bis(benzyloxy)-5,7-dideoxy-(+)-thioepicatechin* (**17**). To a cold solution of **15** (1.42 g, 2.7 mmol, 1 eq.) in CH_2_Cl_2_ (50 mL) was slowly added TFA (494 μL, 2.4 eq.). The resulting mixture was kept at 0 °C. The reaction was quenched by addition of Et_3_N (890 μL, 2.4 eq.) followed by cold H_2_O (15 mL). The organic layer was separated and washed with brine solution (15 mL), dried (Na_2_SO_4_), filtered, and the solvent removed *in vacuo*. The crude product was then purified by silica gel chromatography (10–30% EtOAc in heptane) to produce the desired major diastereomer **16** (830 mg, 68%) and the minor diastereomer **17** (60 mg, 4.9%).

*3’,4’-Bis(benzyloxy)-5,7-dideoxy-(+)-thiocatechin* (**16**). [α]^20^_D _= +108.9 (*c 1*, acetone); ^1^H-NMR (CDCl_3_) δ = 1.98 (d, 2H, *J* = 4.8 Hz), 2.52 (dd, 1H, H_4a_, *J* = 8.4, 7.3 Hz), 3.08 (dd, 1H, H_4b_, *J* = 4, 3.9 Hz), 4.16–4.3 (m, 1H), 5.1 (s, 2H), 5.16 (s, 2H), 6.85–7.2 (m, 6H), 7.22–7.5 (m, 11H); ^13^C-NMR (CDCl_3_) δ = 36.6, 51.7, 56.4, 70.1, 71.3, 77.5, 115.2, 121.8, 124.6, 124.8, 127.3, 127.5, 127.9, 127.9, 128.5, 128.5, 130.5, 130.9, 131.2, 131.9, 132.7, 136.9, 137.5, 148.4, 149.2; Optical purity = 96% *ee*; Chemical purity = 100% (AUC); MS (*m/z*) = 455.1 (M^+^+1), 437.2 (M^+^-OH), 345.3 (M^+^-OH-Bn); HRMS calcd for C_29_H_26_O_3_S [M+H] 455.1681, Found: 455.1677.

*3’,4’-Bis(benzyloxy)-5,7-dideoxy-(+)-thioepicatechin* (**17**). [α]^20^_D _= +38.6 (*c 1*, Acetone); ^1^H NMR (300 MHz, CDCl_3_) δ = 2.2 (d, 1H, *J* = 9.4 Hz), 2.96 (dd, 1H, H_4a_, *J* = 5, 17 Hz), 3.1 (dd, 1H, H_4b_, *J* = 3.5, 17 Hz), 4.3–4.42 (m, 1H), 4.45 (d, 1H, *J* = 1.6 Hz), 5.12 (s, 2H), 5.14 (s, 2H), 6.85–7.2 (m, 6H), 7.28–7.5 (m, 11H); ^13^C NMR (75 MHz, CDCl_3_) δ = 37.9, 50.1, 66.4, 71.3, 76.6, 114.9, 115.6, 121.7, 124.8, 125.9, 126.8, 127.3, 127.5, 127.8, 128.5, 128.5, 130.1, 131.2, 131.4, 132.4, 137.1, 137.3, 148.9, 148.9; Optical purity = 100% *ee*; HPLC purity = 100% (AUC); MS (m/z) = 455.1 (M^+^+1), 437.2 (M^+^-OH), 345.3 (M^+^-OH-Bn); HRMS calcd for C_29_H_26_O_3_S [M+H] 455.1681, Found: 455.1679.

*(1R,2R)-1-(3’,4’-Bis(benzyloxy)phenyl)-3-(2-(tert-butylthio)phenyl)propane-1,2-diol* (**18**). A suspension of AD-mix-β (18 g) in ^t^BuOH/H_2_O (60 mL, 1/1, v/v) was stirred at RT for 15 minutes until a clear yellow solution was obtained. This was followed by the addition of a solution of **14** (3.6g, 7.29 mol, 1 eq.) in CH_2_Cl_2_ (15 mL). The resulting reaction mixture cooled to 0 °C with stirring. Then, MeSO_2_NH_2_ (0.833 g, 8.76 mmol, 1.2 eq.) was added and the mixture stirred at this temperature for 24 h. The reaction was quenched by the addition of 10% Na_2_S_2_O_3_ (50 mL) and then extracted with CH_2_Cl_2_ (2 × 100 mL). The combined organic layers were washed with H_2_O (2 × 50 mL), brine (1 × 75 mL), dried (Na_2_SO_4_), filtered, and the solvent removed *in vacuo* to afford the crude product. The crude product was purified by silica gel chromatography using 20% to 30% EtOAc/heptane to produce **18** (3.2g, 83%) as an oil with 99.8% AUC purity. [α]^20^_D _= -34.3 (*c 1*, acetone); ^1^H-NMR (CDCl_3_) δ = 1.15 (s, 9H), 2.21 (d, 1H, *J = *4.5 Hz), 2.82 (dd, 1H, *J = *9, 9.5 Hz), 2.88 (d, 1H, *J = *4.5 Hz), 3.07 (dd, 1H, *J = *3.4, 4 Hz), 3.81-3.9 (m, 1H), 4.4 (dd, 1H, *J = *3.4, 4 Hz), 5.13 (s, 2H), 5.18 (s, 2H), 6.8 (s, 2H), 7.03 (s, 1H), 7.1–7.52 (m, 14H); ^13^C-NMR (CDCl_3_) δ = 30.9, 38.6, 47.6, 71.4, 71.5, 113.9, 115.1, 120.3, 126.7, 127.3, 127.5, 127.8, 127.8, 128.5, 129.2, 130.9, 132.4, 134.8, 137.3, 137.4, 139.1, 143.8, 148.8, 149.2; Optical purity = 100% *ee*; HRMS calcd for C_33_H_36_O_4_S [M+H] 529.2412, Found: 529.2337.

*3’,4’-Bis(benzyloxy)-5,7-dideoxy-(-)-thiocatechin* (**19**) *and 3’,4’-bis(benzyloxy)-5,7-dideoxy-(-)-thio-epicatechin* (**20**). To a cold solution of **18** (1.6 g, 3.04 mmol, 1 eq.) in CH_2_Cl_2_ (60 mL) was slowly added TFA (617 μL, 3.0 eq.). The resulting mixture was kept at 0 °C. The reaction was quenched by addition of Et_3_N (1.1 mL, 2.4 eq.) followed by cold H_2_O (25 mL). The organic layer was separated and washed with brine solution (25 mL), dried (Na_2_SO_4_), filtered, and the solvent removed *in vacuo*. The crude product was then purified by silica gel chromatography (10–30% EtOAc in heptane) to produce the desired major diastereomer **19** (925 mg, 68%), and minor diastereomer **20** (72 mg, 4.8%).

*3’,4’-Bis(benzyloxy)-5,7-dideoxy-(-)-thiocatechin* (**19**). [α]^20^_D _= -110.4 (*c 1*, acetone); ^1^H-NMR (CDCl_3_) δ = 1.98 (d, 1H, *J = *4.3 Hz), 2.9 (dd, 1H, H_4a_, *J = *8.3, 14.2, 17 Hz), 3.08 (dd, 1H, H_4b_, *J = *4, 16.5 Hz), 4.15–4.32 (m, 2H), 5.12 (s, 2H), 5.16 (s, 2H), 6.8–7.15 (m, 6H), 7.2–7.5 (m, 11H); ^13^C-NMR (CDCl_3_) δ = 31.8, 51.6, 70.1, 71.3, 71.4, 77.2, 115.1, 115.3, 121.8, 124.5, 125.3, 126.8, 127.3, 127.5, 127.9, 127.9, 128.1, 128.5, 128.5, 130.5, 130.9, 131.9, 132.7, 136.9, 137.1, 148.1, 148.1; Optical purity = 99% *ee*; Chemical purity = 100% (AUC); MS (*m/z*) = 455.1 (M^+^+1), 345.3 (M^+^-OH-Bn); HRMS calcd for C_29_H_26_O_3_S [M+H] 455.1681, Found: 455.1678.

*3’,4’-Bis(benzyloxy)-5,7-dideoxy-(-)-thioepicatechin* (**20**). [α]^20^_D _= -74.2 (*c 1*, acetone); ^1^H-NMR (CDCl_3_) δ = 2.2 (d, 1H, *J = *9.4Hz), 2.95 (dd, 1H, H_4a_, *J = *5, 17 Hz), 3.1 (dd, 1H, H_4b_, *J = *3.5, 17 Hz), 4.3-4.41 (m, 1H), 4.42 (d, 1H, *J = *1.4 Hz), 5.1 (s, 2H), 5.12 (s, 2H), 6.8–7.2 (m, 6H), 7.28–7.5 (m, 11H); ^13^C-NMR (CDCl_3_) δ = 37.9, 50.1, 66.4, 71.3, 76.6, 114.9, 115.6, 121.7, 124.8, 125.9, 126.7, 127.2, 127.9, 127.8, 128.0, 128.5, 128.5, 130.1, 131.2, 131.4, 132.4, 137.1, 137.3, 148.9, 148.9; Optical purity = 99.5% *ee*; Chemical purity = 100% (AUC); MS (*m/z*) = 455.1 (M^+^+1), 345.3 (M^+^-OH-Bn); HRMS calcd for C_29_H_26_O_3_S [M+H] 455.1681, Found: 455.1679.

*5,7-Dideoxy-(+)-thiocatechin* (**1**). To an ice cold solution of compound **16** (614 mg, 1.35 mmol, 1 eq.) in dry CH_2_Cl_2_ (50 mL) was added *N,N-*dimethylaniline (1.37 mL, 10.82 mol, 8 eq.) under N_2_. The solution was stirred at this temperature for ~10 minutes before AlCl_3_ (1.8 g, 13.52 mmol, 10 eq.) was added in one portion. The flask was covered with aluminum foil to protect it from light and stirred at ice bath temperature for 3h. EtOAc (100 mL) and silica gel (8.5 g) were added to the reaction mixture and stirred for 15 minutes. The reaction mixture was filtered through a pad of silica gel. The silica gel pad was washed with EtOAc (5 × 50 mL). The filtrates were combined and the solvent was removed *in vacuo* to ~5 mL of volume keeping the bath temperature < 25 °C. The crude mixture was diluted with purified water (50 mL), frozen and lyophilized to give the crude product. The crude product was further purified by preparative HPLC to give the desired compound **1** (180mg, 54%, 100% AUC) as an off-white solid. [α]^20^_D _= + 22.2 (*c 1*, acetone); ^1^H-NMR (acetone-d_6_) δ = 2.9 (dd, 1H, *J = *9, 16.1 Hz), 3.1 (dd, 1H, *J = *4, 16.2 Hz), 4.1 – 4.35 (m, 2H), 6.7 (s, 2H, Ar), 6.9 (s, 1H, Ar), 6.92–7.2 (m, Ar, 4H), 7.4–8.3 (br s, 2H); ^13^C-NMR (acetone-d_6_) δ = 39.2, 52.7, 70.9, 116.0, 116.5, 121.3, 124.9, 125.7, 127.3, 130.9, 131.7, 134.3, 134.8, 145.6, 145.9; MS= 255.2 [M^+^-H_2_O-H, 100%]; HRMS calcd for C_15_H_14_O_3_S [M+H] 275.0742, Found: 275.0737.

*5,7-Dideoxy-(-)-thioepicatechin* (**2**). To an ice cold solution of compound **17** (790 mg, 1.74 mmol, 1 eq.) in dry CH_2_Cl_2_ (80 mL) was added *N,N-*dimethylaniline (1.76 mL, 13.92 mol, 8 eq.) under N_2_. The solution was stirred at this temperature for ~10 minutes before AlCl_3_ (2.32 g, 17.4 mmol, 10 eq.) was added in one portion. The flask was covered with aluminum foil to protect it from light and stirred at the ice bath temperature for 3h. EtOAc (50 mL) and silica gel (10 g) were added to the reaction mixture and stirred for 15 minutes. The reaction mixture was filtered through a pad of silica gel. The silica gel pad was washed with EtOAc (4 × 50 mL). The filtrates were combined and the solvent removed *in vacuo* to ~20 mL of volume keeping the bath temperature < 25 °C. The crude mixture was diluted with purified water (100 mL), cooled and lyophilized to give the crude product. The crude product was further purified by preparative HPLC to give the desired compound **2** (270 mg, 64%, 98.5% AUC) as an off-white solid. [α]^20^_D _= -28.6 (*c 1*, acetone); ^1^H-NMR (acetone-d_6_) δ = 2.88 (dd, 1H, *J = *5.9 and 16.7 Hz), 3.04 (d, 1H, *J = *14.5 Hz), 4.25–4.4 (m, 2H), 6.5–7.2 (m, 7H, Ar), 7.4–8.3 (br s, 2H); ^13^C-NMR (acetone-d_6_) δ = 37.9, 49.3, 66.4, 114.5, 116.1, 120.5, 123.8, 124.9, 126.1, 130.7, 130.9, 131.1, 133.3, 144.3, 144.4; MS= 255.2 [M^+^-H_2_O-H, 100%]; HRMS calcd for C_15_H_14_O_3_S [M+H] 275.0742, Found: 275.0739.

*5,7-Dideoxy-(-)-thiocatechin* (**3**). To an ice cold solution of compound **19** (506 mg, 1.11 mmol, 1 eq.) in dry CH_2_Cl_2_ (40 mL) was added *N, N-*dimethylaniline (1.13 mL, 8.92 mol, 8 eq.) under N_2_. The solution was stirred at this temperature for ~10 minutes before AlCl_3_ (1.5 g, 11.14 mmol, 10 eq.) was added in one portion. The flask was covered with aluminum foil to protect it from light and stirred at the ice bath temperature for 2.5h. EtOAc (100 mL) and silica gel (8 g) were added to the reaction mixture and stirred for 15 minutes. The reaction mixture was filtered through a pad of silica gel. The silica gel pad was washed with EtOAc (3 × 50 mL). The filtrates were combined and the solvent was removed *in vacuo* to ~20 mL of volume keeping the bath temperature < 25 °C. The crude mixture was diluted with purified water (50 mL), cooled and lyophilized to give the crude product. The crude product was further purified by preparative HPLC to give the desired compound **3** (180 mg, 59%, 99% AUC) as an off-white solid. [α]^20^_D _= -21.3 (*c 1*, acetone); ^1^H-NMR (acetone-d_6_) δ = 2.9 (dd, 1H, *J = *9, 16.1 Hz), 3.1 (dd, 1H, *J = *4, 16.2 Hz), 4.1–4.35 (m, 2H), 6.7 (s, 2H, Ar), 6.9 (s, 1H, Ar), 6.92-7.2 (m, Ar, 4H), 7.4–8.3 (br s, 2H); ^13^C-NMR (acetone-d_6_) δ = 39.2, 52.7, 70.9, 116.0, 116.5, 121.3, 124.9, 125.7, 127.3, 130.9, 131.7, 134.3, 134.8, 145.6, 145.9; MS = 255.2 [M^+^-H_2_O-H, 100%]; HRMS calcd for C_15_H_14_O_3_S [M+H] 275.0742, Found: 275.0735.

*5,7-Dideoxy-(+)-thioepicatechin* (**4**). To an ice cold solution of compound **20** (790 mg, 1.74 mmol, 1 eq.) in dry CH_2_Cl_2_ (80 mL) was added *N, N-*dimethylaniline (1.76 mL, 13.92 mol, 8 eq.) under N_2_. The solution was stirred at this temperature for ~10 minutes before AlCl_3_ (2.32 g, 17.4 mmol, 10 eq.) was added in one portion. The flask was covered with aluminum foil to protect it from light and stirred at the ice bath temperature for 3 h. EtOAc (50 mL) and silica gel (10 g) were added to the reaction mixture and stirred for 15 minutes. The reaction mixture was filtered through a pad of silica gel. The silica gel pad was washed with EtOAc (4 × 50 mL). The filtrates were combined and the solvent was removed *in vacuo* to ~20 mL of volume keeping the bath temperature < 25 °C. The crude mixture was diluted with purified water (100 mL), cooled and lyophilized to give the crude product. The crude product was further purified by preparative HPLC to give the desired compound **4** (270mg, 64%, 98.5% AUC) as an off-white solid. [α]^20^_D _= +28.4 (*c 1*, acetone); ^1^H-NMR (acetone-d_6_) δ = 2.88 (dd, 1H, *J = *5.9 and 16.7 Hz), 3.04 (d, 1H, *J = *14.5 Hz), 4.25–4.4 (m, 2H), 6.5–7.2 (m, 7H, Ar), 7.4–8.3 (br s, 2H); ^13^C-NMR (acetone-d_6_) δ = 37.9, 49.3, 66.4, 114.5, 116.1, 120.5, 123.7, 124.9, 126.1, 130.7, 130.9, 131.1, 133.3, 144.3, 144.4; MS = 255.2 [M^+^-H_2_O-H, 100%]; HRMS calcd for C_15_H_14_O_3_S [M+H] 275.0742, Found: 275.0736.

*(E)-1-(2,4-Bis(benzyloxy)-6-hydroxyphenyl)-3-(3’,4’-bis(benzyloxy)phenyl)prop-2-en-1-one* (**22**). To a cold solution (<5 °C) of NaH (60% dispersion in oil, 0.69 g, 17.23 mmol, 1.2 eq.) in DMF (50 mL) was added **21** (5 g, 14.36 mmol, 1 eq.) keeping the internal temperature < 5 °C throughout the addition. The resulting mixture was allowed to stir at this temperature for 20 minutes before addition of a solution of **10** (4.57g, 14.36 mmol, 1 eq.) in DMF (75 mL) over 15 minutes. The resulting mixture was allowed to warm to RT and stirred for 16 h. A gel was obtained. Water (75 mL) and EtOAc (75 mL) were added with stirring whereupon a solid started to appear. Heptane (200 mL) was then added, the solids were suction filtered and washed with heptane (2 × 50 mL). The solids were dried under high vacuum at 40–45 °C for 24 h to produce **22** (8.6g, 92%) as a yellow solid with 100% AUC purity. ^1^H- NMR (CDCl_3_) δ = 4.93 (s, 2H), 5.06 (s, 2H), 5.09 (s, 2H), 5.2 (s, 2H), 6.2 (dd, 2H, *J = *1.6 and 4.6 Hz), 6.6–6.8 (m, 2H), 6.9 (s, 1H), 7.2–7.42 (m, 25H), 7.71 (q, 2H, *J = *15.5 Hz); ^13^C-NMR (CDCl_3_) δ = 70.3, 70.8, 70.9, 71.4, 96.2, 97.8, 105.8, 114.1, 114.9, 123.1, 125.3, 126.9 (2C), 127.3 (2C), 127.2 (2C), 127.6 (2C), 127.8, 127.9, 128.0, 128.3 (2C), 128.4, 128.5 (4C), 128.6 (2C), 136.2, 136.3, 136.8, 136.9, 142.8, 149.5, 149.6, 161.1, 164.7, 166.3, 191.5. Anal. calcd for C_43_H_36_O_6_, C 79.61, H 5.59 Found C 79.58, H 5.36.

*(E)-3,5-Bis(benzyloxy)-2-(3’,4’-bis(benzyloxy)phenyl)allyl)phenol* (**23**). To a solution of ethanol (236 mL) and THF (800 ml) was added CeCl_3_.7H_2_O (74 g, 198.0 mmol, 2.5 eq.) at room temperature and the mixture was stirred at this temperature until a clear solution was obtained. To this was added chalcone **22** (51.4 g, 79.23 mmol, 1 eq.) followed by THF (500 mL). The solution was stirred at room temperature for ~10 minutes and then cooled to −1.5 to −0.2 °C (internal temperature) with agitation. Solid NaBH_4_ (7.5 g, 197.37 mmol, 2.5 eq.) was added in portions over 0.5 h keeping the internal temperature ≤ 0.3 °C throughout the addition. The mixture was stirred at this temperature (−0.8 to −0.3 °C) for ~2.5 h. The reaction mixture was quenched with 5% aqueous citric acid (167 mL) followed by EtOAc (1.5 L). The mixture was stirred as the internal temperature rose to ~12 °C. The organic layer was separated and washed with H_2_O (2 × 1L, 1 × 800 mL), brine (1 × 500 mL), dried (Na_2_SO_4_), filtered and the solvent was removed *in vacuo* to give a semi solid. HPLC analysis of the crude product indicated 86% product and 14% by-product (AUC). The crude product was purified by silica gel chromatography using heptane/CH_2_Cl_2_/EtOAc (25/25/0.5, v/v/v) to give **23** (38g, 76%) as an-off white solid with 99.5% AUC purity. ^1^H-NMR (CDCl_3_) δ = 3.55 (d, *J = *5.4 Hz, 2H), 4.94–5.08 (m, 5H), 5.12 (d, *J = *4.4 Hz, 4H), 6.04–6.2 (m, 2H), 6.22–6.4 (m, 2H), 6.82 (s, 2H), 6.97 (d, *J = *1.2 Hz, 1H), 7.18–7.5 (m, 20H); ^13^C-NMR (CDCl_3_) δ = 26.4, 70.2, 70.4, 71.5, 93.6, 95.3, 107.0, 112.9, 115.3, 119.9, 126.7, 127.3, 127.3, 127.4, 127.5, 127.8, 127.8, 127.9, 128.0, 128.5, 128.6, 128.5, 128.6, 130.9, 136.5, 137.3, 137.4, 146.6, 148.2, 155.8, 157.9, 158.8. Anal. calcd for C_43_H_38_O_5_, C 81.36, H 6.03, Found C 81.22, H 5.86.

*(E)-3,5-Bis(benzyloxy)-2-(3’,4’-bis(benzyloxy)phenyl)allylphenyl trifluoromethane sulfonate* (**24**). To an ice cold solution of **23** (6 g, 9.45 mmol, 1 eq.) in pyridine (35 mL) was slowly added Tf_2_O (1.75 mL, 10.4 mmol, 1.1 eq). The resulting mixture was allowed to warm to RT and stirred for 18h. The reaction mixture was concentrated *in vacuo* and the residue was dissolved in EtOAc (250 mL) and washed with 1N HCl (2 × 100 mL). The aqueous layer was back washed with EtOAc (100 mL). The organic layers were combined and washed with brine (2 × 75 mL), dried (Na_2_SO_4_), filtered and the solvent removed *in vacuo* to produce the crude product. The crude product was purified by silica gel chromatography (10–15% EtOAc in heptane) to produce the **24** (4.1 g, 82%) as an off-white solid with 100% AUC purity. ^1^H-NMR (CDCl_3_) δ = 3.55 (d, 2H, *J* = 6.4 Hz), 5.0 (s, 2H), 5.05 (s, 2H), 5.08 (s, 2H), 5.1 (s, 2H), 5.95–6.1 (m, 1H), 6.28 (d, 1H, *J* = 16 Hz), 6.5 (d, 1H, *J* = 2.3 Hz), 6.6 (d, 1H, *J* = 2.3 Hz), 6.7–6.86 (m, 2H), 6.9 (d, 1H, *J *= 1.8 Hz), 7.3–7.5 (m, 20H); ^13^C-NMR (CDCl_3_) δ = 70.7, 71.5, 77.4, 100.4, 115.3, 125.1, 127.3, 127.4, 127.5, 127.6, 128.19, 128.5, 125.7, 128.8. Anal. calcd for C_44_H_37_F_3_O_7_S, C 68.92, H 4.86, F 7.43 Found C 68.89, H 4.77, F 7.22.

*(E)-(3,5-Bis(benzyloxy)-2-(3-(3’,4’-bis(benzyloxy)phenyl)allyl)phenyl) (tert-butyl)sulfane* (**25**). To a degassed solution of triflate **24** (1.86 g, 2.4 mmol, 1 eq.) in toluene (50 mL) was added Pd(OAc)_2_ (33 mg, 0.14 mmol, 0.06 eq.) and (*R*)-Tol-BINAP (115 mg, 0.17 mmol, 0.07 eq.) at room temperature. The mixture was again degassed for an additional 15 minutes at room temperature. In a separate flask, KN(TMS)_2_ (0.5M solution in toluene, 7 mL, 3.4 mmol, 1.4 eq.) was added to *t-*BuSH (0.39 mL) followed by stirring the mixture at RT for 15 minutes before adding this solution to the mixture of triflate containing catalyst. The resulting reaction mixture was kept at 100–105 ºC (bath temperature) for 36 h. The reaction was quenched by adding H_2_O (50 mL) and extracted with EtOAc (2 × 100 mL). The combined organic layers were washed with H_2_O (50 mL), brine (50 mL), dried, (Na_2_SO_4_), filtered and the solvent removed *in vacuo* to give the crude product. The crude product was then purified by silica gel chromatography (20–40% CH_2_Cl_2_ in heptane) to afford **25** (1.25 g, 73%) as an off-white solid with 98.3% AUC purity; ^1^H-NMR (CDCl_3_) δ = 1.28 (s, 9H), 3.85 (d, 2H, *J* = 5.9 Hz), 4.98 (s, 2H), 5.01 (s, 2H), 5.06 (s, 2H), 5.12 (s, 2H), 6.05–6.25 (m, 2H), 6.6 (d, 1H, *J* = 2.4 Hz), 6.7–6.85 (m, 3H), 6.9 (d, 1H, *J *= 1.8 Hz), 7.2–7.5 (m, 20H); ^13^C-NMR (CDCl_3_) δ = 31.3, 31.6, 47.4, 70.3, 70.3, 71.5, 71.6, 77.5, 101.8, 112.7, 113.4, 115.5, 119.7, 127.1, 127.4, 127.5, 127.5, 127.7, 127.7, 127.9, 128.1, 128.4, 128.4, 128.5, 128.6, 129.6, 132.3, 133.8, 136.9, 137.5, 139.5, 148.1, 149.2, 156.1, 157.1; MS (m/z) = 707.1 (M^+^+1), 651.2 (M^+^-^t^Bu). Anal. calcd for C_47_H_46_O_4_S, C 79.85, H 6.56, Found C 79.64, H 6.49.

*(1S,2S)-(3-(2,4-Bis(benzyloxy)-6-(tert-butylthio)phenyl)-1-(3’,4’-bis(benzyloxy)phenyl)propane-1,2-diol* (**26**). To a solution of *^t^*BuOH/H_2_O (1/1, v/v, 100 mL) was added AD-mix-α (16.5 g) and the suspension was stirred at RT until a clear solution was obtained. The solution was then cooled to 0-5 ºC and a solution of **25** (3.3 g, 4.67 mmol) in CH_2_Cl_2_ (100 mL) was added in one portion followed by CH_3_SO_2_NH_2_ (0.533 g, 5.6 mol, 1.2 eq.). The resulting reaction mixture was stirred at this temperature for 24 h. After 24h and 30h, additional amounts of AD-mix-α (16.5 g) and CH_3_SO_2_NH_2_ (0.533 g, 5.6 mol, 1.2 eq.) were added respectively and the stirring was continued for an additional 24h at this temperature. HPLC analysis indicated the completion of the reaction. To the reaction mixture was added H_2_O (100 ml) and EtOAc (150 mL). The organic layer was separated and washed with 10% aqueous sodium metabisulfite (2 x 100 mL), H_2_O (100 mL), brine (50 mL), dried (Na_2_SO_4_), filtered and the solvent removed *in vacuo* to afford the crude product as an off-white solid. The solid was triturated with heptane (50 mL) at room temperature and filtered to give **26** (3.48 g, 100%) as an off-white solid with 97% AUC purity; [α]^20^_D _= -17.4 (*c* 1, acetone); Optical purity = 95% *ee; *^1^H-NMR (CDCl_3_) δ = 0.2 (s, 6H), 1.2 (s, 9H), 2.4 (d, 2H, *J *= 6 Hz), 3.1 (d, 2H, *J* = 6.05 Hz), 3.15 (s, 1H), 3.8–3.95 (m, 1H), 4.35 (d, 1H, *J* = 4.5 Hz), 4.96 (s, 2H), 5.0 (s, 2H), 5.05 (s, 2H), 6.65 (d, 1H, *J* = 2.4 Hz), 6.75–6.9 (m, 2H), 7.05 (d, 1H, *J* = 1.4 Hz), 7.25–7.5 (m, 20H); ^13^C-NMR (CDCl_3_) δ = 31.1, 41.9, 70.3, 70.8, 71.4, 71.6, 77.5, 102.1, 115.2, 116.1, 120.2, 125.2, 127.3, 127.4, 127.5, 127.5 127.7, 128.1, 128.2, 129.4, 128.4, 128.7, 128.8, 134.5, 135.3, 137.4, 149.1; MS (m/z) = 723.1 (M^+^-OH). Anal. calcd for C_47_H_48_O_6_S, C 76.19, H 6.53, Found C 75.98, H 6.44.

*(1R,2R)-(3-(2,4-Bis(benzyloxy)-6-(tert-butylthio)phenyl)-1-(3’,4’-bis(benzyloxy)phenyl)propane-1,2-diol* (**30**). To a solution of *^t^*BuOH/H_2_O (1/1, v/v, 90 mL) was added AD-mix-β (6.1 g) and the suspension was stirred at RT until a clear solution was obtained. The solution was then cooled to 0–5 ºC and a solution of **25** (1.22 g, 1.73 mmol) in CH_2_Cl_2_ (60 mL) was added in one portion followed by CH_3_SO_2_NH_2_ (0.197 g, 2.07 mol, 1.2 eq.). The resulting reaction mixture was stirred at this temperature for 24 h and the progress of the reaction monitored by HPLC. After 24 h and 30 h, additional amounts of AD-mix-α (6.1 g) and CH_3_SO_2_NH_2_ (0.197 g, 2.07 mol, 1.2 eq.) were added respectively and stirring continued for an additional 24h at this temperature. To the reaction mixture was added H_2_O (50 mL) and EtOAc (100 mL). The organic layer was separated and washed with 10% aqueous sodium metabisulfite (2 × 100 mL), H_2_O (50 mL), brine (50 mL), dried (Na_2_SO_4_), filtered and the solvent removed *in vacuo* to afford the crude product as an off-white solid. The solid was purified by silica gel chromatography (30% EtOAc in heptane) to give **30** (1.2 g, 98%) as an off-white solid with 99.4% AUC purity. [α]^20^_D _= +16.4 (*c* 1, acetone); Optical purity =95% *ee*; ^1^H-NMR (CDCl_3_) δ = 0.2 (s, 6H), 1.2 (s, 9H), 2.4 (d, 1H, *J = *6.2 Hz), 3.05 (d, 2H, *J = *6.2 Hz), 3.15 (d, 1H, *J = *3.1 Hz), 3.75–3.82 (m, 1H), 4.3–4.4 (m, 1H), 4.95 (s, 2H), 5.0 (s, 2H), 5.1 (s, 4H), 6.6 (d, 1H, *J = *2.4 Hz), 6.7–6.85 (m, 2H), 7.0 (s, 1H), 7.15–7.5 (m, 20H); ^13^C-NMR (CDCl_3_) δ = 31.1, 41.9, 70.3, 70.8, 71.4, 71.6, 77.5, 102.1, 115.2, 116.1, 120.2, 125.2, 127.3, 127.4, 127.5, 127.5, 127.7, 128.1, 128.2, 129.4, 128.4, 128.7, 128.8, 134.5, 135.3, 137.4, 149.1; MS (m/z) = 723.1 (M^+^-OH). Anal. calcd for C_47_H_48_O_6_S, C 76.19, H 6.53, Found C 76.11, H 6.39.

*5,7,3’,4’-Tetra-O-benzyl-(+)-thiocatechin* (**27**) *and 5,7,3’,4’-Tetra-O-benzyl-(+)-thioepicatechin* (**28**). To a cold solution (0–5 °C) of **26** (3.46 g, 4.68 mmol, 1 eq.) in CH_2_Cl_2_ (150 mL) was added TFA (1.28 g, 11.22 mmol, 2.4 eq.). The mixture was kept at 0-5 °C for 24 hours. Upon consumption of the starting material, the reaction was quenched by addition of Et_3_N (1.5 mL) and cold H_2_O (50 mL). The organic layer was separated and washed with brine (50 mL), dried (Na_2_SO_4_), filtered and the solvent removed *in vacuo*. The crude product was then purified by silica gel chromatography (heptane/CH_2_Cl_2_/EtOAc, 25/25/1, v/v/v) to afford **27** (1.75 g, 57%), and **28** (440 mg, 14%).

*5,7,3’,4’-Tetra-O-benzyl-(+)-thiocatechin* (**27**). [α]^20^_D _= +55.2 (*c* 1, CH_2_Cl_2_); Chemical purity = 97% (AUC); Optical purity = 96% *ee*; ^1^H-NMR (CDCl_3_) δ = 2.0 (d, 1H, *J* = 5.5 Hz), 2.7 (dd, 1H, *J* = 8.4, 17.2 Hz), 3.1 (dd, 1H, *J *= 4.5, 17.2 Hz), 4.1 (d, 1H, *J* = 8.5 Hz), 4.15-4.3 (m, 1H), 4.95 (s, 4H), 5.1 (s, 2H), 5.12 (s, 2H), 6.4 (s, 2H), 6.85–6.95 (m, 2H), 7.01 (d, 1H, *J* = 1.5 Hz), 7.2–7.5 (m, 20H); ^13^C-NMR (CDCl_3_) δ = 29.8, 31.9, 51.3, 70.0, 70.2, 70.3, 71.3, 71.4, 77.5, 97.4, 103.3, 113.8, 115.2, 115.4, 121.9, 127.3, 127.5, 127.6, 127,9, 127.9, 127.9, 128.1, 128.5, 128.5, 128.6, 128.6, 130.62, 133.9, 136.8, 136.8, 137.0, 137.2, 148.1, 149.2, 157.1, 158.1; MS (*m/z*) = 667.5 (M^+^+1). Anal. calcd for C_43_H_38_O_5_S, C 77.45, H 5.74, S 4.81 Found C 77.36, H 5.59, S 4.62.

*5,7,3’,4’-Tetra-O-benzyl-(+)-thioepicatechin* (**28**). [α]^20^_D _= +61.3 (*c* 1, CH_2_Cl_2_); Optical purity = 96.6% *ee*; Chemical purity = 100% (AUC); ^1^H-NMR (CDCl_3_) δ = 2.2 (d, 1H, *J* = 9.6 Hz), 2.9 (q, AB_q_, *J_A_* = 3.9, 4.1, 17.4 Hz, *J_B _* = 4.9, 5.3 Hz), 4.3–4.42 (m, 2H), 4.95 (s, 2H), 5.12 (s, 2H), 5.14 (s, 2H), 6.3 (s, 2H), 6.82 (d, 1H, *J* = 8.3 Hz), 6.98 (dd, 1H, *J *= 2, 8.3 Hz), 7.12 (d, 1H, *J *= 2 Hz), 7.2–7.5 (m, 20H); ^13^C-NMR (CDCl_3_) δ = 31.4, 31.9, 49.8, 66.4, 70.0, 70.3, 71.3, 71.4, 77.5, 97.7, 102.3, 112.3, 114.9, 115.65, 121.7, 127.2,. 127.3, 127.5, 127.6, 127.8, 127.9, 128.1, 128.5, 128.5, 128.6, 128.6, 131.4, 133.7, 136.8, 136.8, 137.2, 137.3, 148.4, 148.9, 158.1, 158.5; MS (*m/z*) = 667.5 (M^+^+1). Anal. calcd for C_43_H_38_O_5_S, C 77.45, H 5.74, S 4.81 Found C 77.28, H 5.63, S 4.66.

*5,7,3’,4’-Tetra-O-benzyl-(-)-thiocatechin* (**31**) *and 5,7,3’,4’-tetra-O-benzyl-(-)-thioepicatechin* (**32**). To a cold solution (0–5 °C) of **30** (1.26 g, 1.62 mmol, 1 eq.) in CH_2_Cl_2_ (60 mL) was added TFA (300 μL, 3.89 mmol, 2.4 eq.). The mixture was kept at 0–5 °C. Upon consumption of the starting material, the reaction was quenched by addition of Et_3_N (515 μL) and cold H_2_O (25 mL). The organic layer was separated and washed with brine (25 mL), dried (Na_2_SO_4_), filtered and the solvent removed *in vacuo*. The crude product was then purified by silica gel chromatography (heptane/CH_2_Cl_2_/EtOAc, 1/1/0 to 25/25/1, v/v/v) to afford the desired compounds **31** (0.82 g, 76%), and **32** (170 mg, 16%).

*5,7,3’,4’-Tetra-O-benzyl-(-)-thiocatechin* (**31**). [α]^20^_D_= -62.5 (*c* 1, CH_2_Cl_2_); Chemical purity = 98.6% (AUC); Optical purity = 96% *ee*; ^1^H-NMR (CDCl_3_) δ = 1.95 (d, 1H, J = 4.4 Hz), 2.7 (dd, 1H, *J* = 8.4, 17.2 Hz), 3.12 (dd, 1H, *J* = 4.5, 17.2 Hz), 4.15 (d, 1H, *J* = 8.5 Hz), 4.18–4.32 (m, 1H), 4.98 (s, 4H), 5.1 (s, 2H), 5.12 (s, 2H), 6.3 (s, 2H), 6.8–6.92 (m, 2H), 7.02 (d, 1H, *J* = 1.5 Hz), 7.2–7.5 (m, 20H); ^13^C-NMR (CDCl_3_) δ = 29.8, 51.3, 70.0, 70.2, 70.3, 71.3, 71.4, 77.5, 97.4, 102.3, 113.8, 115.4, 121.9, 127.3, 127.5, 127.6, 127.9, 127.9, 127.9, 128.1, 128.2, 128.5, 128.5, 128.6, 128.6, 129.1, 130.6, 133.9, 136.8, 136.9, 137., 137.2, 149.2, 149.2, 157.8, 159.0; MS (*m/z*) = 667.5 (M^+^+1). Anal. calcd for C_43_H_38_O_5_S, C 77.45, H 5.74, S 4.81 Found C 77.41, H 5.66, S 4.78.

*5,7,3’,4’-Tetra-O-benzyl-(-)-thioepicatechin* (**32**). [α]^20^_D_= -58.4 (*c* 1, CH_2_Cl_2_); Chemical purity = 100% (AUC); Optical purity = 97% *ee*; ^1^H-NMR (CDCl_3_) δ = 2.2 (d, 1H, J = 10.5 Hz), 2.9 (ABq, *J_A_* = 4, 17.7 Hz, *J_B_* = 5, 17.9 Hz), 4.3–4.42 (m, 2H), 4.92 (s, 4H), 5.06 (s, 2H), 5.09 (s, 2H), 6.36 (s, 2H), 6.86 (d, 1H, *J *= 8.3 Hz), 6.95 (dd, 1H, *J* = 2.1, 8.3 Hz), 7.12 (d, 1H, *J* = 2 Hz), 7.2–7.5 (m, 20H); ^13^C-NMR (CDCl_3_) δ= 29.8, 51.3, 70.0, 70.2, 70.3, 71.3, 71.4, 76.6, 97.4, 102.3, 113.8, 115.2, 115.4, 121.9, 127.3, 127.5, 127.6, 127.9, 127.9, 127.9, 128.1, 128.2, 128.5, 128.5, 128.6, 128.6, 129.1, 130.6, 133.9, 136.8, 136.8, 137.0, 137.2, 140.2, 140.2, 157.3, 158.0; MS (*m/z*) = 667.5 (M^+^+1). Anal. Calcd for C_43_H_38_O_5_S, C 77.45, H 5.74, S 4.81 Found C 77.37, H 5.65, S 4.58.

*(+)-Thiocatechin* (**5**). To an ice cold solution of **27** (1.43 g, 2.15 mmol, 1 eq.) in dry CH_2_Cl_2_ (60 mL) was added *N,N-*dimethylaniline (1.63 mL, 12.88 mol, 8 eq.) under N_2_. The solution was stirred at this temperature for ~10 minutes before AlCl_3_ (2.29 g, 17.18 mmol, 10 eq.) was added in one portion. The flask was covered with aluminum foil to protect it from light and stirred at ice bath temperature for 2 h. EtOAc (50 mL) and silica gel (5 g) were added to the reaction mixture and stirred for 5 minutes. The reaction mixture was filtered through a pad of silica gel. The silica gel pad was washed with EtOAc (5 × 50 mL). The filtrates were combined and the solvent was removed *in vacuo* to ~10 mL of volume, keeping the bath temperature < 25 °C. The crude mixture was diluted with purified water (50 mL), cooled and lyophilized to give the crude product. The crude product was further purified by preparative HPLC to give **5** (380 mg, 57%) as an off-white solid in 99% AUC purity. [α]^20^_D _= +32.2 (*c* 1, Acetone); ^1^H-NMR (acetone-d_6_) δ = 2.58 (dd, 1H, J = 8.3, 15.5 Hz), 3.18 (d, 1H, *J* = 16.3 Hz), 4.17 (d, 1H, *J *= 8.3 Hz), 4.32 (s, 1H), 6.08 (s, 1H), 6.2 (s, 1H), 6.8 (s, 2H), 6.92 (s, 1H); ^13^C-NMR (acetone-d_6_) δ = 32.3, 52.3, 71.1, 99.9, 103.6, 112.3, 116.0, 116.5, 121.4, 131.3, 135.7, 145.6, 145.8, 157.0; MS = 307.1 [M^+^+H]; HRMS Calcd for C_15_H_15_O_5_S [M+H] 307.0642 Found 307.0644.

*(-)-Thioepicatechin* (**6**). To an ice cold solution of **32** (0.67 g, 1.01 mmol, 1 eq.) in dry CH_2_Cl_2_ (35 mL) was added *N,N-*dimethylaniline (1.02 mL, 8.05 mol, 8 eq.) under N_2_. The solution was stirred at this temperature for ~10 minutes before AlCl_3_ (1.37 g, 10.1 mmol, 10 eq.) was added in one portion. The flask was covered with aluminum foil to protect it from light and stirred at the ice bath temperature for 2 h. EtOAc (50 mL) and silica gel (5 g) were added to the reaction mixture and stirred for 5 minutes. The reaction mixture was filtered through a pad of silica gel. The silica gel pad was washed with EtOAc (3 × 50 mL). The filtrates were combined and the solvent was removed *in vacuo* to ~10 mL of volume keeping the bath temperature < 25 °C. The crude mixture was diluted with purified water (50 mL), cooled and lyophilized to give the crude product. The crude product was further purified by preparative HPLC to give **6** (100 mg, 57%) as an off-white solid in 99.8% AUC purity. [α]^20^_D_= -28.9 (*c *1, acetone); ^1^H-NMR (acetone-d_6_) δ = 2.7 (dd, 1H, *J* = 6.7, 17 Hz), 2.82 (dd, 1H, *J* = 4.1, 17 Hz), 4.32 (d, 1H, *J* = 2.3 Hz), 4.33–4.5 (br s, 1H), 6.16 (dd, 2H, *J* = 2.3, 11.8 Hz), 6.72 (d, 1H, *J* = 8 Hz), 6.82 (dd, 1H, *J *= 2.3, 11.8 Hz), 7.08 (d, 1H, *J* = 2 Hz), 7.7–8.3 (br s, 4H); ^13^C-NMR (acetone-d_6_) δ = 50.0, 68.0, 99.9, 104.1, 110.6, 115.5, 117.1, 121.7, 133.0, 135.2, 145.3, 145.4, 156.9, 157.5; MS = 307.1 [M^+^+H]; HRMS Calcd for C_15_H_15_O_5_S [M+H] 307.0642 Found 307.0634.

*(-)-Thiocatechin* (**7**). To an ice cold solution of **31** (0.92 g, 1.38 mmol, 1 eq.) in dry CH_2_Cl_2_ (40 mL) was added *N, N-*dimethylaniline (1.08 mL, 8.5 mol, 6.2 eq.) under N_2_. The solution was stirred at this temperature for ~10 minutes before AlCl_3_ (1.51 g, 11 mmol, 8.2 eq.) was added in one portion. The flask was covered with aluminum foil to protect it from light and stirred at the ice bath temperature for 2 h. EtOAc (50 mL) and silica gel (5 g) were added to the reaction mixture and stirred for 5 minutes. The reaction mixture was filtered through a pad of silica gel. The silica gel pad was washed with EtOAc (3 × 50 mL). The filtrates were combined and the solvent was removed *in vacuo* to ~10 mL of volume keeping the bath temperature < 25 °C. The crude mixture was diluted with purified water (50 mL), cooled and lyophilized to give the crude product. The crude product was further purified by preparative HPLC to give **7** (205 mg, 56%) as an off-white solid in 99% AUC purity. [α]^20^_D_= -28.6 (*c* 1, acetone); ^1^H-NMR (acetone-d_6_) δ = 2.58 (dd, 1H, *J* = 5.9, 16.7 Hz), 3.18 (dd, 1H, *J* = 4.7, 16.7 Hz), 3.8–3.96 (br s, 1H), 4.1 (d, 1H, *J *= 9 Hz), 4.16–4.3 (m, 1H), 6.12 (d, 1H, *J* = 2.2 Hz), 6.2 (d, 1H, *J* = 2.2 Hz), 6.72 (s, 2H, *J* = 0.8 Hz), 6.9 (s, 1H), 7.6–8.3 (br s, 4H); ^13^C-NMR (acetone-d_6_) δ = 32.4, 52.3, 71.1, 98.5, 103.5, 112.0, 115.9, 116.5, 121.4, 131.3, 135.8, 145.5, 145.8, 157.0; MS = 307.1 [M^+^+H]; HRMS Calcd for C_15_H_15_O_5_S [M+H] 307.0642 Found 307.0633.

*(+)-Thioepicatechin* (**8**). To an ice cold solution of **28** (0.4 g, 0.6 mmol, 1 eq.) in dry CH_2_Cl_2_ (20 mL) was added *N,N-*dimethylaniline (0.6 mL, 4.81 mol, 8 eq.) under N_2_. The solution was stirred at this temperature for ~10 minutes before AlCl_3_ (0.8 g, 6.01 mmol, 10 eq.) was added in one portion. The flask was covered with aluminum foil to protect it from light and stirred at the ice bath temperature for 2 h. EtOAc (50 mL) and silica gel (5 g) were added to the reaction mixture and stirred for 5 minutes. The reaction mixture was filtered through a pad of silica gel. The silica gel pad was washed with EtOAc (3 × 50 mL). The filtrates were combined and the solvent was removed *in vacuo* to ~10 mL of volume keeping the bath temperature < 25 °C. The crude mixture was diluted with purified water (50 mL), cooled and lyophilized to give the crude product. The crude product was further purified by preparative HPLC to give **8** (124 mg, 52%) as an off-white solid in 100% AUC purity. [α]^20^_D_= +29.7 (*c* 1, acetone); ^1^H-NMR (acetone-d_6_) δ = 2.7 (dd, 1H, *J *= 6.7, 17 Hz), 2.82 (dd, 1H, *J *= 4.1, 17 Hz), 4.32 (d, 1H, *J* = 2.3 Hz), 4.33-4.5 (br s, 1H), 6.16 (dd, 2H, *J* = 2.3, 11.8 Hz), 6.72 (d, 1H, *J *= 8 Hz), 6.82 (dd, 2H, *J* = 2.3, 11.8 Hz), 7.08 (d, 1H, *J *= 2 Hz), 7.7–8.3 (br s, 4H); ^13^C-NMR (acetone-d_6_) δ = 50.0, 68.0, 99.9, 104.1, 110.6, 115.5, 117.1, 121.7, 133.0, 135.2, 145.3, 145.4, 156.9, 157.5; MS = 307.1 [M^+^+H]; HRMS Calcd for C_15_H_15_O_5_S [M+H] 307.0642 Found 307.0631.

## 4. Conclusions

In conclusion, the first enantioselective syntheses of sulfur analogues of naturally occurring flavan-3-ols were accomplished wherein the oxygen atom of the pyran ring has been replaced with a sulfur atom in a stereoselective fashion. The key steps involved were:the introduction of a –S*t*-Bu group via Pd(0) chemistry, stereoselective dihydroxylation under Sharpless conditions, ring closure under acidic conditions via the formation of benzylic carbocation, and the removal of benzyl group using AlCl_3_ and *N,N-*dimethylaniline as a scavenger. The enantiomeric excess of the intermediates and the title compounds were determined by a chiral HPLC method. The methodologies developed here are quite modular and should permit the preparation of other analogues of flavan-3-ols and various combinations of A-type and B-type proanthocyanidins [48,49].
